# Feasibility of the *Transport PLUS* intervention to improve the transitions of care for patients transported home by ambulance: a non-randomized pilot study

**DOI:** 10.1186/s40814-022-01138-5

**Published:** 2022-08-05

**Authors:** Kevin G. Munjal, Sai Kaushik Yeturu, Hugh H. Chapin, Nadir Tan, Diana Gregoriou, Daniela Garcia, Corita Grudzen, Ula Hwang, Barbara Morano, Hayley Neher, Ksenia Gorbenko, Glen Youngblood, Anjali Misra, Staley Dietrich, Cyndi Gonzalez, Giselle Appel, Erica Jacobs, Albert Siu, Lynne D. Richardson

**Affiliations:** 1 Department of Emergency Medicine, Icahn School of Medicine at Mount Sinai, 1 Gustave L. Levy Place, Box 1620, New York, NY 10029 USA; 2 Department of Emergency Medicine, New York University School of Medicine, New York, NY USA; 3 Department of Emergency Medicine, Yale University School of Medicine, New Haven, CT USA; 4 Institute for Health Equity Research and Department of Population Health Science and Policy, Icahn School of Medicine at Mount Sinai, New York, NY USA; 5 Sidney Kimmel Medical College, Philadelphia, PA USA; 6 George Washington University School of Medicine, Washington D.C., USA; 7 Department of Geriatrics, Icahn School of Medicine at Mount Sinai, New York, NY USA

**Keywords:** Emergency medical technicians, Prehospital care, Community paramedicine, Mobile integrated healthcare, Discharge comprehension, Fall safety, Emergency medical services, Readmissions, Transitions of care

## Abstract

**Background:**

The growing population of patients over the age of 65 faces particular vulnerability following discharge after hospitalization or an emergency room visit. Specific areas of concern include a high risk for falls and poor comprehension of discharge instructions. Emergency medical technicians (EMTs), who frequently transport these patients home from the hospital, are uniquely positioned to aid in mitigating transition of care risks and are both trained and utilized to do so using the *Transport PLUS* intervention.

**Methods:**

Existing literature and focus groups of various stakeholders were utilized to develop two checklists: the fall safety assessment (FSA) and the discharge comprehension assessment (DCA). EMTs were trained to administer the intervention to eligible patients in the geriatric population. Using data from the checklists, follow-up phone calls, and electronic health records, we measured the presence of hazards, removal of hazards, the presence of discharge comprehension issues, and correction or reinforcement of comprehension. These results were validated during home visits by community health workers (CHWs). Feasibility outcomes included patient acceptance of the *Transport PLUS* intervention and accuracy of the EMT assessment. Qualitative feedback via focus groups was also obtained. Clinical outcomes measured included 3-day and 30-day readmission or ED revisit.

**Results:**

One-hundred three EMTs were trained to administer the intervention and participated in 439 patient encounters. The intervention was determined to be feasible, and patients were highly amenable to the intervention, as evidenced by a 92% and 74% acceptance rate of the DCA and FSA, respectively. The majority of patients also reported that they found the intervention helpful (90%) and self-reported removing 40% of fall hazards; 85% of such changes were validated by CHWs. Readmission/revisit rates are also reported.

**Conclusions:**

The *Transport PLUS* intervention is a feasible, easily implemented tool in preventative community paramedicine with high levels of patient acceptance. Further study is merited to determine the effectiveness of the intervention in reducing rates of readmission or revisit. A randomized control trial has since begun utilizing the knowledge gained within this study.

## Key messages regarding feasibility

- What uncertainties existed regarding the feasibility?
  Prior to this pilot study, *Transport PLUS* was a novel intervention that had not previously been implemented or attempted. It was unknown whether prehospital providers could be easily trained and whether the intervention could be operationalized within an EMS agency. There was also uncertainty regarding the prehospital providers’ capacity to accurately complete the assessment and whether patients would be amenable to receiving the intervention, particularly if they would be accepting of EMS providers performing the discharge comprehension assessment and in-home fall safety assessments. Specific items to be included in the relevant surveys and the training format/delivery also required development and feedback.
- What are the key feasibility findings?
  Training providers using a combination of in-person practical and online didactic training was feasible and effective. To address high provider turnover, the study team developed online asynchronous learning tools; however, the in-person practical remained necessary to ensure competency and fidelity of the intervention. Dispatch was found to be capable of assigning appropriate units when patients were eligible. Patients were highly amenable to the intervention, though with some reservations on specific items of the fall safety assessment. Community health worker validation resulted in high reliability of reported findings. Finally, paper checklists utilized in this study were difficult to integrate into the patient’s electronic health records.
- What are the implications of the feasibility findings for the design of the main study?
  These positive feasibility findings of patient acceptance and provider capacity provided sufficient basis with which to design a subsequent randomized control trial to further evaluate the intervention. The feasibility findings directly informed improvements to the checklist and to the training program to be used in the main study. Items of the fall safety assessment, particularly high-reach items in cabinets, were modified in the main study to be a question as opposed to a visual survey, thereby addressing patient and provider concerns about discomfort in searching patient home cabinets. The checklists utilized in the interventions were also digitized, resolving the troubles of transmitting data from the intervention by paper.

## Background

Adults aged 65 and older accounted for approximately 23 million emergency department (ED) visits in the USA in 2016 [1], a number expected to continue to grow as the population of adults in this age group is anticipated to double over the next 40 years [2]. These patients are especially vulnerable following a hospitalization, which is often associated with functional decline. One study demonstrated that 40% of adults aged 60 or older will face a fall in 6 months following a hospitalization, and over half of these result in injury [3]. It is also known that older adult patients face a statistically significant increased risk of 30-day readmission to the emergency department (ED) when transported home by ambulance [4]. The frequent and diverse utilization of healthcare services by the elderly requires attention to risks in transition of care and lapses of patient education, particularly as they transition from hospital environment to home. Two key risks are addressed in this demonstration: falls in the home and comprehension of discharge instructions.

Older adults face a variety of comorbidities and serious risk of injury due to falls which may result in hip fractures, head injuries, and other serious trauma. The fall-related mortality rate for older adults increased by 30% from 2007 to 2016. The same report indicates that falls among the elderly are not only serious but also a costly public health concern, comprising 50 billion USD in 2015 healthcare spending with Medicare and Medicaid covering 75% of those costs [5]. *Transport PLUS* offers a unique opportunity to deliver an in-home fall prevention intervention targeted to high-risk patients transported home by ambulance following hospital or ED discharge.

Meanwhile, patient discharge instructions are often dense with information and difficult for patients to comprehend. Competing priorities among patients, caregivers, and providers can contribute to confusion, and time pressures often leave little opportunity for the patient to obtain clarity before leaving the hospital. In one study of ED discharge instruction comprehension, at all ages, it was found that 78% of patients have a deficiency in their understanding of their own aftercare. More alarmingly, among this same cohort of patients, deficient comprehension was only recognized by the patient 20% of the time where it was demonstrated, highlighting that patients frequently are unaware when they fail to understand their aftercare [6]. Lack of comprehension in discharge care is prevalent outside of the ED as well, with adults 65 and older discharged from medical and surgical units demonstrating noncomprehension of recommended follow-up appointments (5%), medications (27%), exercise (48%), and diet (50%) [7]. For patients transported home by ambulance, this is an opportunity to improve understanding of discharge instructions.

Emergency medical services (EMS), while still most commonly recognized for 9-1-1 emergency response, are already being utilized to transport patients home by ambulance. More can be accomplished by these medically trained professionals, and they have the capacity to directly improve the patient’s transition of care. EMS provides transportation of particularly vulnerable patients to their home setting from both hospital inpatient stays, as well as from the ED. As previously mentioned, among older patients, those transported home by ambulance are particularly vulnerable [4].

The evolving practice, often referred to as “community paramedicine” or “mobile integrated healthcare,” seeks to expand the role of emergency medical technicians (EMTs) and paramedics to help support patients’ needs in the home and in the community. The intent of these programs is to prevent emergencies in the community, thereby decreasing the present burden on crowded emergency departments. *Transport PLUS*, a collaboration between the Mount Sinai Health System and partner commercial ambulance agencies, was designed to train and utilize EMTs as a valuable member of the continuum of high-quality healthcare delivery. EMTs were trained to perform a discharge comprehension assessment and a home fall safety assessment for patients over the age of 65 and their families or caregivers during routine transports to the home after being discharged from the ED or from inpatient units.

In this paper, we describe the implementation of the *Transport PLUS* intervention and present the results of a feasibility study to evaluate whether EMTs were able to successfully perform the intervention and whether patients found the intervention helpful.

## Methods

The *Transport PLUS* program is a novel EMS transition of care intervention that trains EMTs who are already transporting older adult patients (65+ years in age) home from a single, urban academic hospital by ambulance. The study was conducted between November 2013 and July 2014. EMTs were trained to perform two simple interventions: a home fall safety assessment (FSA) and a discharge comprehension assessment (DCA). Patients transitioning to other hospitals, nursing homes, or any other institution providing formal post-discharge care were excluded from the study. Both the FSA and DCA were developed using a checklist developed through a comprehensive review of the literature and existing tools.

### Development of the *Transport PLUS* intervention

**Fig. 1:**
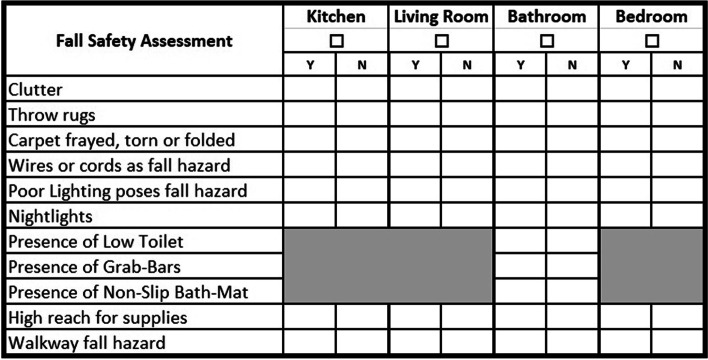
Fall safety assessment checklist

The home FSA is a brief scan of the home or apartment for easily recognized fall hazards. The extensive literature search, which included publications from the fields of nursing and physical therapy, generated a long list of fall hazards potentially present in the home [8–13]. Given that the *Transport PLUS* intervention was to be administered by EMTs, and not overly extend the amount of time the EMTs were spending in the home, we developed a simple, brief, and manageable checklist. The fall hazards selected for the checklist were those that are common, easily assessed with a high degree of inter-rater reliability, modifiable by the patient or their caregiver, and most likely to make an impact on patients’ risk of falls in the home. For example, structural hazards which would require home renovation or significant financial expenditure were omitted. Consensus among the multidisciplinary study team on which items accomplished those goals resulted in the 11-item list shown in Fig. 1. The study team was multidisciplinary and consisted of physicians, nurses, social workers, and care managers.

**Fig. 2:**
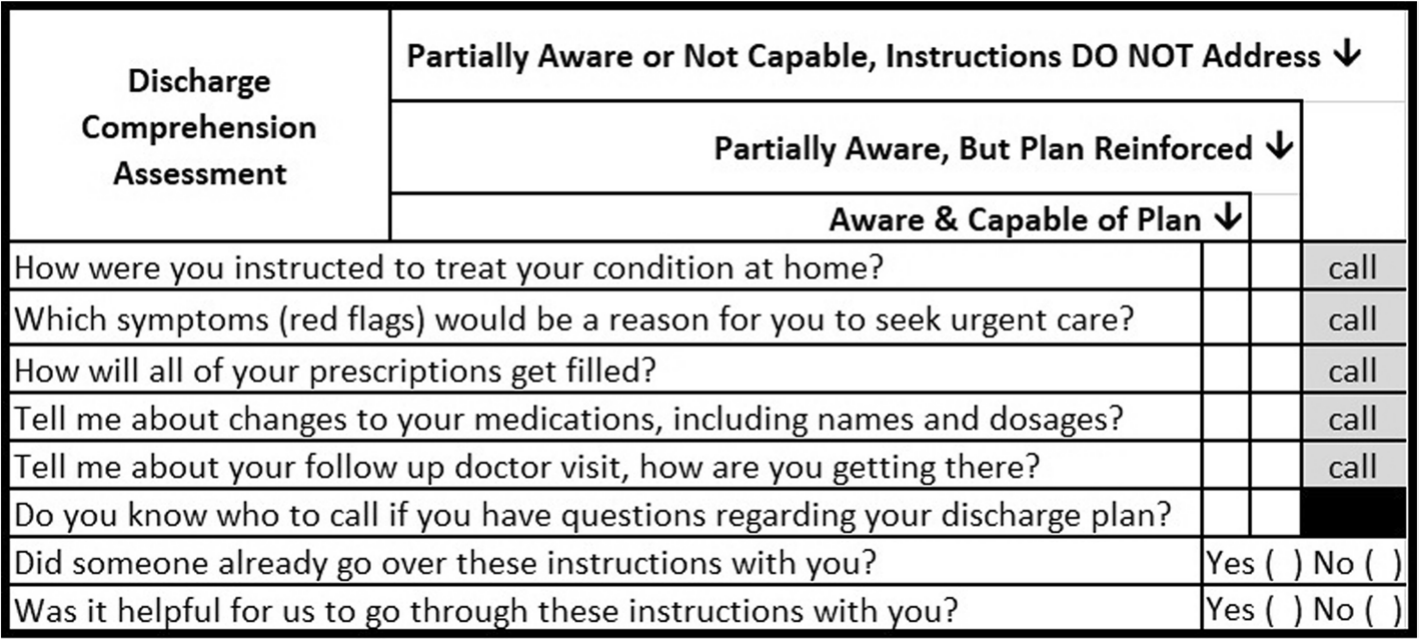
Discharge comprehension assessment checklist

The DCA is a structured conversation between the EMT and the patient (and their caregiver, when present in the ambulance) that occurs during the routine transportation encounter (beginning from the patient’s hospital bed to assisting them into their home). The checklist was developed utilizing Coleman’s four “pillars” of a patient-centered transition of care: medication self-management, use and maintenance of a patient-centered record, primary care and specialist follow-up, and knowledge of red flags [14]. Our adapted checklist assesses the patient’s understanding of and access to medications, follow-up appointments, transportation, and self-care instructions (Fig. 2). Specific items were again determined by study team consensus on adherence to the Coleman pillars. The checklist provides areas for EMTs to assess or reinforce patient awareness of plan. When EMTs were unable to correct awareness or other issues arose, they were instructed to call the multidisciplinary study team for further guidance.

### EMT training

EMT training sessions for the assessments were administered to optimize effectiveness and inter-rater reliability. This training included a 1-h didactic session (offered either in-person or through an asynchronous online module which was developed during the study), as well as a 1-h practical component that included two simulations of obtaining consent and administering the *Transport PLUS* intervention. Training was iterative, and EMT feedback was used in continually refining its development. The didactic training included, in addition to the checklists, the importance of patient comprehension of discharge instructions and how to review sample discharge paperwork while completing the DCA checklist. Samples of actual discharge paperwork given to patients/caregivers upon discharge were used for this purpose. Pre and post testing was administered, and participation in the intervention required a post-test score of 85%.

### The *Transport PLUS* intervention administration and outcome measures

To determine the feasibility of *Transport PLUS*, patient acceptance of the *Transport PLUS* intervention and accuracy of the EMT assessment were assessed. Data was collected on the frequency of fall hazards identified, fall hazard removal or correction, EMT-identified deficiencies in discharge comprehension, and correction or reinforcement to discharge comprehension. Clinical outcomes measured included 3-day and 30-day readmission. Secondary outcomes include patient-reported helpfulness of the intervention, qualitative focus group results, and CHW home visit to validate EMT assessments.

*Transport PLUS* was performed for all patients that accepted the intervention, and all completed checklists were collected. Patients were asked verbally for permission to make a 4-week follow-up phone call, during which patients were asked to self-report the presence of fall hazards in their home and if previously identified hazards by the EMTs had been removed or addressed. Finally, after the study was completed, some additional patient feedback was obtained in the form of a focus group to qualitatively assess patient response to the intervention. Patients were asked questions about their comfort with the intervention, prompted for concerns with the program, and given the opportunity to provide any additional feedback.

A limited number of randomly selected patients were given the option for a follow-up visit with a community health worker (CHW), who was also trained in identifying home fall hazards. Each CHW judged the validity of the initial EMT assessment of reported fall hazards. Aggregate percentages of overall removal of fall hazards were collected to validate patient self-reported data and the EMT’s initial assessment. Finally, a review of the electronic health record was performed to yield readmission or ED revisit at 3- and 30-day post-intervention. This study was reviewed and approved by the Institutional Review Board of the Icahn School of Medicine at Mount Sinai.

## Results

The *Transport PLUS* program trained a total of 103 EMTs, all of whom successfully completed the course and were eligible to deliver the *Transport PLUS* intervention. Four-hundred thirty-nine encounters were made where patients were offered the *Transport PLUS* intervention. These encounters consisted of 327 unique patients. Unique patients are considered for demographic data, whereas all other results are reported relative to total encounters. Demographic information is provided in Table 1.

**Table 1:** Patient demographics (*n* = 326)

	Count	Percent
Age (years)		
65–69	38	12%
70–79	78	24%
80–89	131	40%
90–99	73	22%
> 100	6	2%
Sex		
F	214	66%
M	112	34%
Race		
White	119	37%
Black	94	29%
Hispanic	66	20%
Asian	7	2%
Native American	1	0%
Unavailable	39	12%
Insurance		
Private	135	41%
Medicare	317	97%
Medicaid	182	56%
Dual eligible	178	55%

The first outcome of interest is patient acceptability of the intervention, which is reported separately for DCA and FSA. DCA was accepted in 404 patient encounters (92.03%). FSA was accepted in 323 patient encounters (73.58%). Among these, 3 returned checklists were only partially completed, whereas the remaining FSA and all DCA checklists were completed in full.

EMTs identified a total of 2117 fall hazards over the course of the study. Of these, 1094 unique hazards were identified. (If in a given encounter there were multiple hazards of the same type identified [e.g., throw rugs], all are counted in total hazard statistics reported but would only count as one unique identification.) Four-week follow-up phone surveys then yielded hazard removal rates as reported by the patient. Phone surveyors were able to reach patients regarding 316 encounters. When asked about hazards documented by the EMT, patients denied or disagreed with the presence of the hazard in 34% of cases. Of the hazards which were acknowledged by the patient, the overall removal rate was 40% statistical data for fall hazards, and follow-up call data per hazard are reported in Tables 2 and 3, respectively.

**Table 2:** Fall hazards

Unique (*n* = 1,094)	
Average	3.398
Median	3
Max	10
Total (*n* = 2,117)	
Average	6.570
Median	6
Max	30

**Table 3:** Hazard removal upon follow-up phone call

	Clutter	Throw rugs	Carpet frayed, torn, or folded	Wires or cords as fall hazard	Poor lighting poses fall hazard	Nightlights absent	Low toilet	Lack of grab bars	Lack of nonslip bath mat	High reach for supplies	Walkway fall hazard	Total
Removals	11	17	6	13	14	13	20	38	30	25	13	200
Hazard remains present	8	22	15	30	52	58	13	55	29	17	6	305
Hazard denial	9	20	14	35	27	36	16	68	16	9	9	259
Refused to answer	22	21	21	21	21	22	21	21	21	24	21	236
Percent of removals among hazards acknowledged	58%	44%	29%	30%	21%	18%	61%	41%	51%	60%	68%	40%

Patient-reported data on hazard removal was validated during home visits by CHWs. Forty such CHW assessments were performed. Among this group, 85.7% of hazards which were self-reported as removed were confirmed to have actually been removed.

DCA results revealed 94 (23%) patients who accepted the DCA had at least one area of DCA deficiency. Data reported on each of the six deficiencies individually is reported in Table 4. Of note, among the 260 total categorical deficiencies found, EMTs were able to reinforce the discharge plan and correct comprehension deficiency with the patient in 199 cases (76.5%). The follow-up survey also found that 90.2% of patients who accepted the *Transport PLUS* intervention and answered the survey question stated that they found the intervention to be helpful. Three-day and 30-day ED revisits and hospital readmissions are categorized by the origin of the initial transport home and reported in Table 5.

**Table 4:** Discharge comprehension (*n* = 404)

	Q1: instructions	Q2: red flags	Q3: fill Rx	Q4: med changes	Q5: follow-up	Q6: who to call
Complete awareness and capability	363	354	375	358	362	352
Partial awareness, plan reinforced	30	40	21	35	33	40
Partial or no awareness, unable to correct	11	10	8	11	9	12

**Table 5:** Returns and readmission

	Return ED visit		Return admission	
	No.	%	No.	%
Transported home from ED (*n* = 136)				
3 days	10	7.35%	7	5.15%
30 days	38	27.94%	23	16.91%
Transported home from inpatient (*n* = 250)				
3 days	13	5.20%	10	4.00%
30 days	75	30.00%	52	20.80%

Revisit and readmission rates reported here are among only those patients who accepted at least one component of the intervention (DCA, FSA, or both) and excludes two patients whose charts were inaccessible for patient privacy

Focus group feedback at the conclusion of the study yielded a few notable constructive comments. For example, some patients expressed discomfort in the assessment of their living space; the cabinet search for high-reach items felt particularly intrusive. Also, some EMTs expressed concern about the additional time in the home that the intervention took. These concerns are further addressed in the discussion.

## Discussion

The results demonstrate that patients are highly amenable to the *Transport Plus* intervention and overwhelmingly found it helpful. EMTs found the intervention feasible to incorporate into their workflow. The preliminary data on return ED visits and readmission confirm that this is a high-risk population. It is a reasonable hypothesis that future studies involving more rigorous methods such as a randomized controlled trial may find a causal relationship between interventions associated with transport home and readmission reduction given the significant rates of deficiency correction in both the FSA and DCA. This hypothesis would be consistent with previous findings that discharge comprehension issues, and post-discharge falls are highly prevalent, which both are linked to readmission, and that patients transported home by ambulance are known to be at high risk for readmission [3, 4, 6].

Several challenges pertaining to the feasibility of the *Transport PLUS* intervention were identified and addressed during this pilot study. Training was initially in-person, but the program experienced high EMS staff turnover rates, leading to a need for online training. Our experience was consistent with EMS literature which has found high EMT turnover to be a national phenomenon with one longitudinal study finding a mean annual turnover rate in EMS agencies of 10.7% [15]. This problem remains unresolved and has been further exacerbated by the global pandemic. Therefore, any training program associated with ambulance personnel must prepare for frequent turnover and be able to provide rapid training to new EMS staff.

Resource-related challenges were also faced in delivering the program. Supervisors were responsible for maintaining fidelity of the program. These supervisors were a limited resource and could only periodically observe visits. It was also envisioned that EMS dispatch would be able to determine when a patient might be eligible and prioritize dispatch of a *Transport PLUS* capable unit. In practice, this was challenging to implement given the numerous other responsibilities that took precedence, such as call acuity, response time, and resource management. Lastly, the checklists were paper forms that needed to be carefully handled and delivered, a problem which was avoided in further study by digitization of the checklists.

Focus group results indicated concerns related to intrusive searches regarding high-reach items in cabinets, as well as concerns from EMTs over the additional minutes spent on the scene. The former was used to inform modifications to the intervention, such as replacing the request for cabinet search with a less obtrusive question asking patients to report if they had any high-reach items of need. EMS operational data was also reviewed to address time concerns, and while call duration times were not available data to be studied here, no notable disruptions to call response were reported due to the additional time spent at the patient’s home.

Our findings were limited by a lack of a comparison group. As the intervention was funded as a demonstration project, it was offered to all qualifying patients with trained providers capable of providing the intervention. Another limitation of the study is that it was limited to patients being discharged from a single urban hospital, and the results may not be generalizable to other patient populations. Lastly, the study is limited by data collected during the 2013–2014 study period due to resource limitations.

We can, however, compare *Transport PLUS* to similar EMS-based interventions in existing literature. Infinger et al. recently reported the development of a reliable survey of environmental risk factors for elderly patients in the prehospital setting. Their content validation procedure ultimately yielded a 9-item checklist with high demonstrated inter-rater reliability. Notable similarities to the list deployed here in the FSA include walkway trip hazards, rugs, clutter, and adequate lighting. Notable inclusions in their tool, which were absent in our intervention, are furniture, slippery floors, and stair condition [16]. It may be worth adjusting the *Transport PLUS* checklist in future iterations to accommodate these important areas of concern.

*Transport PLUS* is yet another step in developing the emerging field of community paramedicine/mobile integrated healthcare (CP/MIH) which aims to reduce emergency utilization of EMS through early recognition and intervention. In the previously discussed Coleman study of transitions of care, nurses, social workers, or other transitional coaches were sent to the home post-discharge to address the pillars; however, this service is not feasible in all communities or for all patients, due to workforce or financial constraints [14]. This inspired the creation of the DCA as part of the *Transport PLUS* intervention to provide increased transitional care efforts to patients who might not receive a home visit. A randomized control trial conducted by Agarwal et al. deployed community paramedicine using validated tools and compared utilization between buildings that received the intervention, termed CP@clinic, and those receiving usual care. Their intervention resulted in a 19% reduction in relative EMS call volume [17]. In yet another study, CP/MIH for a Medicare Advantage population was found to save 2.4 million and results in a 2.97 million (USD) return on investment, further highlighting reduced utilization [18]. While these findings are not directly related to transition of care upon discharge, they add credence to the importance of prevention and education that can be uniquely and effectively administered by EMS providers as is done in this study. A randomized control trial is currently underway evaluating, as primary outcomes, falls occurring in the following 3 months and 3-day and 30-day readmission rates for the *Transport PLUS* intervention [19].

## Conclusions

This study demonstrated the feasibility of the *Transport PLUS* intervention and high acceptability to both patients and EMS providers. Widespread implementation of such an intervention could be readily achieved through the dissemination of existing materials to more providers utilizing the training process reported here. The *Transport PLUS* intervention would be easily scalable and inexpensive to administer. The findings from this pilot study also identified a number of opportunities for improvement to be implemented prior to a future randomized control trial. Further study involving more widespread implementation should also be completed to evaluate generalizability and long-term outcomes pertaining to healthcare utilization.

## Data Availability

The datasets generated and analyzed during this study are available from the corresponding author on a reasonable request.

## Abbreviations

CHW: Community health worker
CP: Community paramedicine
DCA: Discharge comprehension assessment
ED: Emergency department
EMT: Emergency medical technician
FSA: Fall safety assessment
MIH: Mobile integrated healthcare
